# Effects of auxin (indole-3-butyric acid) on growth characteristics, lignification, and expression profiles of genes involved in lignin biosynthesis in carrot taproot

**DOI:** 10.7717/peerj.10492

**Published:** 2020-12-08

**Authors:** Ahmed Khadr, Guang-Long Wang, Ya-Hui Wang, Rong-Rong Zhang, Xin-Rui Wang, Zhi-Sheng Xu, Yong-Sheng Tian, Ai-Sheng Xiong

**Affiliations:** 1 State Key Laboratory of Crop Genetics and Germplasm Enhancement, Ministry of Agriculture and Rural Affairs Key Laboratory of Biology and Germplasm Enhancement of Horticultural Crops in East China, College of Horticulture, Nanjing Agricultural University, Nanjing, China; 2 Department of Horticulture, Faculty of Agriculture, Damanhour University, Damanhour, Egypt; 3 School of Life Science and Food Engineering, Huaiyin Institute of Technology, Huaian, China

**Keywords:** Auxin, *Daucus carota L.*, Gene expression, Growth, Indole-3-butyric acid, Lignification

## Abstract

Carrot is an important root vegetable crop abundant in bioactive compounds including carotenoids, vitamins, and dietary fibers. Carrot intake and its products are gradually growing owing to its high antioxidant activity. Auxins are a class of plant hormones that control many processes of plant growth and development. Yet, the effects of exogenous application of auxin on lignin biosynthesis and gene expression profiles of lignin-related genes in carrot taproot are still unclear. In order to investigate the effect of exogenous indole-3-butyric acid (IBA) on lignin-related gene profiles, lignin accumulation, anatomical structures and morphological characteristics in carrot taproots, carrots were treated with different concentrations of IBA (0, 50, 100, and 150 µM). The results showed that IBA application significantly improved the growth parameters of carrot. The 100 or 150 µM IBA treatment increased the number and area of xylem vessels, whereas transcript levels of lignin-related genes were restricted, resulting in a decline in lignin content in carrot taproots. The results indicate that taproot development and lignin accumulation may be influenced by the auxin levels within carrot plants.

## Introduction

Carrot (*Daucus carota* L.), a biennial vegetable crop belonging to the Apiaceae family, is one of the world’s top ten vegetable crops (Que et al., 2019). Carrot crops are widely cultivated throughout the world, whereas China ranked first in the world in terms of production by 17.9 million tons (FAO, 2018). Carrots are known for their high yield with abundant nutritional components including carotenes and plant fibers. It has high nutritional and medicinal values (Arscott & Tanumihardjo, 2010; Luby, Maeda & Goldman, 2014). The growth and development of carrot taproot directly determines the yield of carrot and quality. It is reported that plant hormones play key roles in the development of carrot taproot (Wang et al., 2015; Khadr et al., 2020).

Plant hormones are involved in various processes including plant growth and development, cell division, cell elongation and differentiation, and apical dominance (Davies, 2013). The root development and nutrient absorption and transportation by roots are directly related to the endogenous hormone levels (Wang et al., 2015). Auxins are a class of phytohormones that control numerous processes of plant growth and development (Davies, 2013). Auxins are known primarily for their ability to induce cell elongation. In addition, they stimulate cell division, vascular differentiation, and root initiation. Indole-3-acetic acid (IAA) is an essential auxin in the plant, which is produced mainly in meristematic tissues of young leaves (Kaya, Tuna & Okant, 2010). IAA and naphthalene acetic acid (NAA) treatment enhanced the formation of tomato fruit (Kaya, Tuna & Okant, 2010). The application of IAA can improve plant height and shoot length (Bose, Jana & Mukhopadhyay, 1980).

Indole-3-butyric acid (IBA) is a kind of auxin that exists naturally in various species of plants and tissues (Epstein & Ludwig-Müller, 2010). IBA may be transformed to IAA and vice versa, suggesting that the metabolism of the two auxins is highly correlated (Woodward & Bartel, 2005). IBA demonstrates the special auxin potential of polar cell-to-cell transfer, but probably through a different mechanism from IAA (Rashotte et al., 2003). IBA is more stable in solutions than IAA and has a much stronger influence on initiation rooting (Nordström, Jacobs & Eliasson, 1991; Ludwig-Müller, 2000). In several bioassays, IBA has auxin activity and affects the rooting process in various plant species more obviously than IAA (Nordström, Jacobs & Eliasson, 1991; Wiesman, Riov & Epstein, 1988; Wiesman, Riov & Epstein, 1989). Furthermore, the IBA application promotes elongation of stems in intact pea plants (Yang & Davies, 1999). Also, IBA stimulates shoot, hypocotyl and lateral root growth (Yang & Davies, 1999; Poupart & Waddell, 2000). IBA could act as an important source of auxin to boost stem elongation in intact plants (Yang & Davies, 1999). However, the root growth and its response to IBA treatment depend on the concentration. A high concentration of IBA (10^−7^ M) inhibited root length in maize and induced the enlargement of root diameter. On the contrary, a lower concentration (10^−9^ M∼10^−12^ M IBA) stimulated root growth and root diameter (Šípošová et al., 2019).

Lignin is the second most abundant biopolymer in vascular plants after cellulose. It is mainly deposited in secondary cell walls and is involved in mechanical support and water transport (Rogers & Campbell, 2004; Zhao & Dixon, 2011). In addition, lignin can respond to environmental stimuli and growth signals including hormone stimuli, pathogen invasion, abiotic stresses, and mechanical attack (Moura et al., 2010; Duan et al., 2019). The excess lignin accumulation in the taproot results in poor quality of carrot (Wang et al., 2017). Therefore, it is important to regulate the lignin levels in the fleshy taproot of carrot.

This study aims to investigate the influence of applied IBA on the anatomy and lignification of carrot taproots. We performed a morphological and anatomical characterization and found that IBA application not only enlarged the taproot diameter but also decreased lignin accumulation in the taproot. Moreover, the transcript levels of genes in lignin biosynthesis and polymerization were altered in response to the IBA treatment. The results could enhance our understanding about the IBA role in taproot growth and lignin accumulation.

## Materials & Methods

### Plant materials

In the present study, the carrot cultivar Kurodagosun was used as plant materials. The carrot seeds were germinated on wet filter paper for 48 h at 25 °C in full darkness for further experiments. The resulting plants were then moved into plastic pots (30 × 27 cm) in a greenhouse and maintained at 22 ± 3 °C and 18 ± 3 °C (day and night, respectively) with 65∼75% humidity under a 14/10 h photoperiod. The plants were grown in a mixture of organic soil and vermiculite (1:1, v/v).

To determine the effect of IBA treatment on carrot taproot growth, different concentrations of IBA solution (0, 50, 100, and 150 µM) (Beijing Solarbio Science & Technology Co., Ltd, Beijing, China) were prepared. After 40 days of sowing, the soil mixture was sprayed with 200 mL of different concentrations of IBA, four times at three-day intervals, whereas soil samples around control plants were sprayed with water. Carrot samples were harvested for analysis at 60 days after sowing.

### Analysis of the anatomical structure of carrot taproot

To examine the changes in carrot taproot structure, cross-sections of treated carrot taproots were harvested. Sample slides were prepared according to the procedure in our published work (Wang et al., 2017). Then, sample slides were analyzed using a light microscope (Olympus brand, CellSens software).

### Quantitative determination of lignin content in carrot taproot

Carrot taproot samples were prepared with three independent biological replicates. Lignin was extracted and quantified according to the method described by Cervilla and his colleagues (Cervilla et al., 2009). The absorption was detected at 280 nm using SpectraMax iD5, Soft Max Pro7 Software (Molecular Devices, Shanghai, China).

### Total RNA extraction and cDNA synthesis

Total RNA was extracted using an RNA extraction kit (Tiangen, Beijing, China). Quantification of total RNA concentration was carried out using a NanoDrop ND-1000 spectrophotometer (NanoDrop Technologies, Wilmington, USA). The total RNA was treated with gDNAEraser (TaKaRa, Dalian, China) for 2 min at 42 °C, to remove the DNA contamination. Synthesis of cDNA from total RNA was carried out using the PrimeScriptRT reagent kit (TaKaRa, Dalian, China).

### Gene selection and expression profiles analysis by RT-qPCR

Genes involved in lignin biosynthesis and polymerization were selected based on a genomic and transcriptomic database CarrotDB (http://apiaceae.njau.edu.cn/carrotdb) (Xu et al., 2014; Wang et al., 2018a). Real time quantitative PCR (RT-qPCR) experiment was performed using TaKaRa SYBR Premix *Ex Taq* to quantify the transcript expression in treated plant samples. The primers of *DcPAL*, *DcC4H*, *Dc4CL*, *DcHCT*, *DcC3′H*, *DcCCoAOMT*, *DcF5H*, *DcCOMT*, *DcCCR*, *DcCAD*, *DcPER1*, *DcLAC1*, and *DcLAC2* used for RT-qPCR assays were selected from our previously published article (Wang et al., 2017). All PCR reaction mixtures consist of 10 µL of SYBR Premix *Ex Taq*, 7.2 µL of deionized water, 0.4 µL of each forward and reversed primer, and 2 µL of tenfold diluted cDNA. PCR cycle was set up to run initially 30 s at 95 °C, followed by 40 cycles at 95 °C for 5 s, and 60 °C for 30 s. *DcActin* was used as internal standard for gene normalization (Tian et al., 2015).

### Statistical analysis

The difference in the mean data of the treatments was analyzed at the 0.05 significance level by Duncan’s method using the SPSS statistics software (Version 16, SPSS Inc. Chicago, USA).

## Results

### Effect of IBA treatment on morphological characteristics of carrot plants

To examine the influence of exogenous IBA on the morphological characteristics of carrot plants, carrot taproots were treated with different concentrations of IBA. Seven morphological parameters including plant length, shoot length, taproot length, shoot fresh weight, taproot fresh weight, number of petioles, and taproot diameter of carrot plants were determined for comparison among the different treatments.

**Figure 1:**
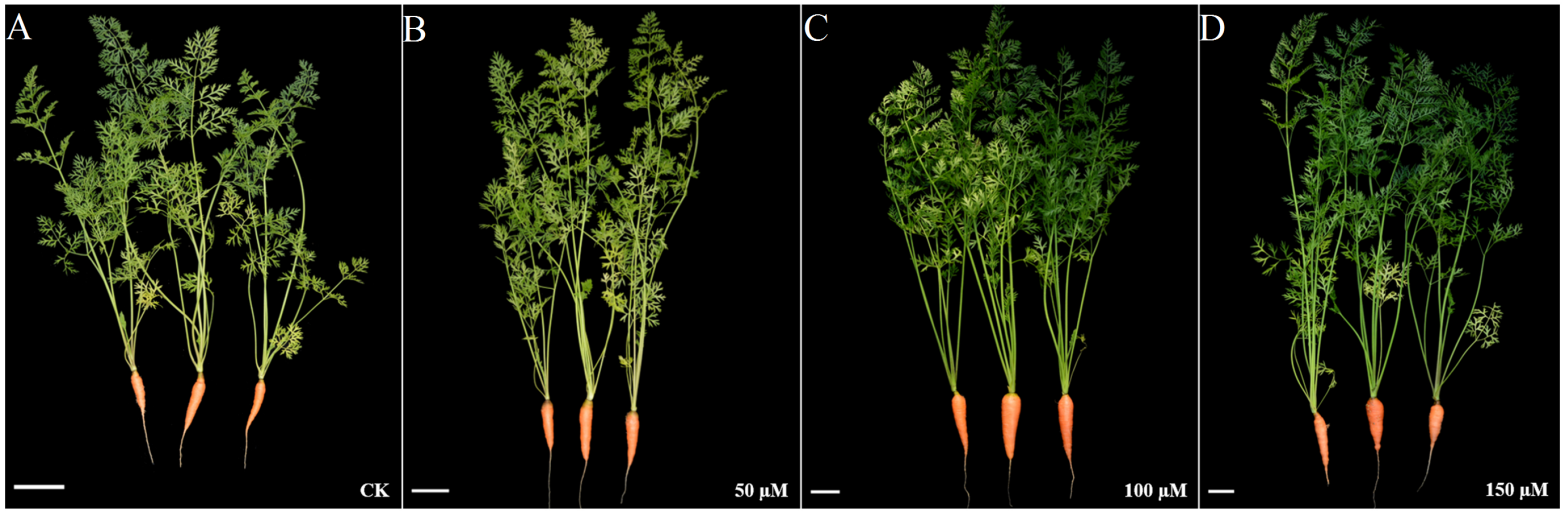
Effect of IBA on the growth status of carrots. Carrot plants treated with 0 (A), 50 (B), 100 (C), and 150 (D) µM IBA were shown. White lines in the lower left corner of each plant represent 5 cm in that pixel.

**Figure 2:**
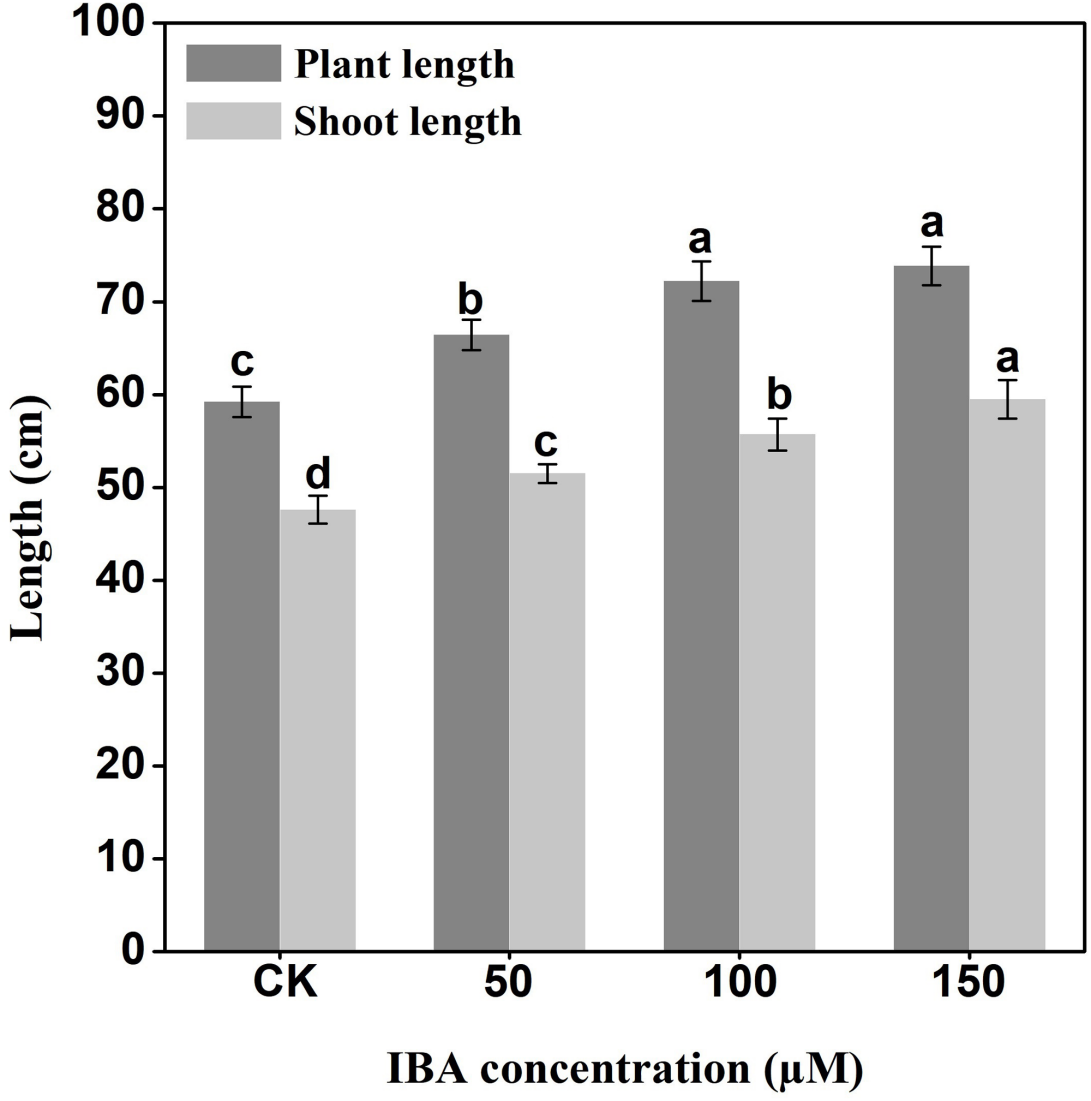
Effect of IBA on overall plant length and shoot length of carrots. Error bars represent standard deviation (SD). Lowercase letters represent significant differences at *P* < 0.05.

**Figure 3:**
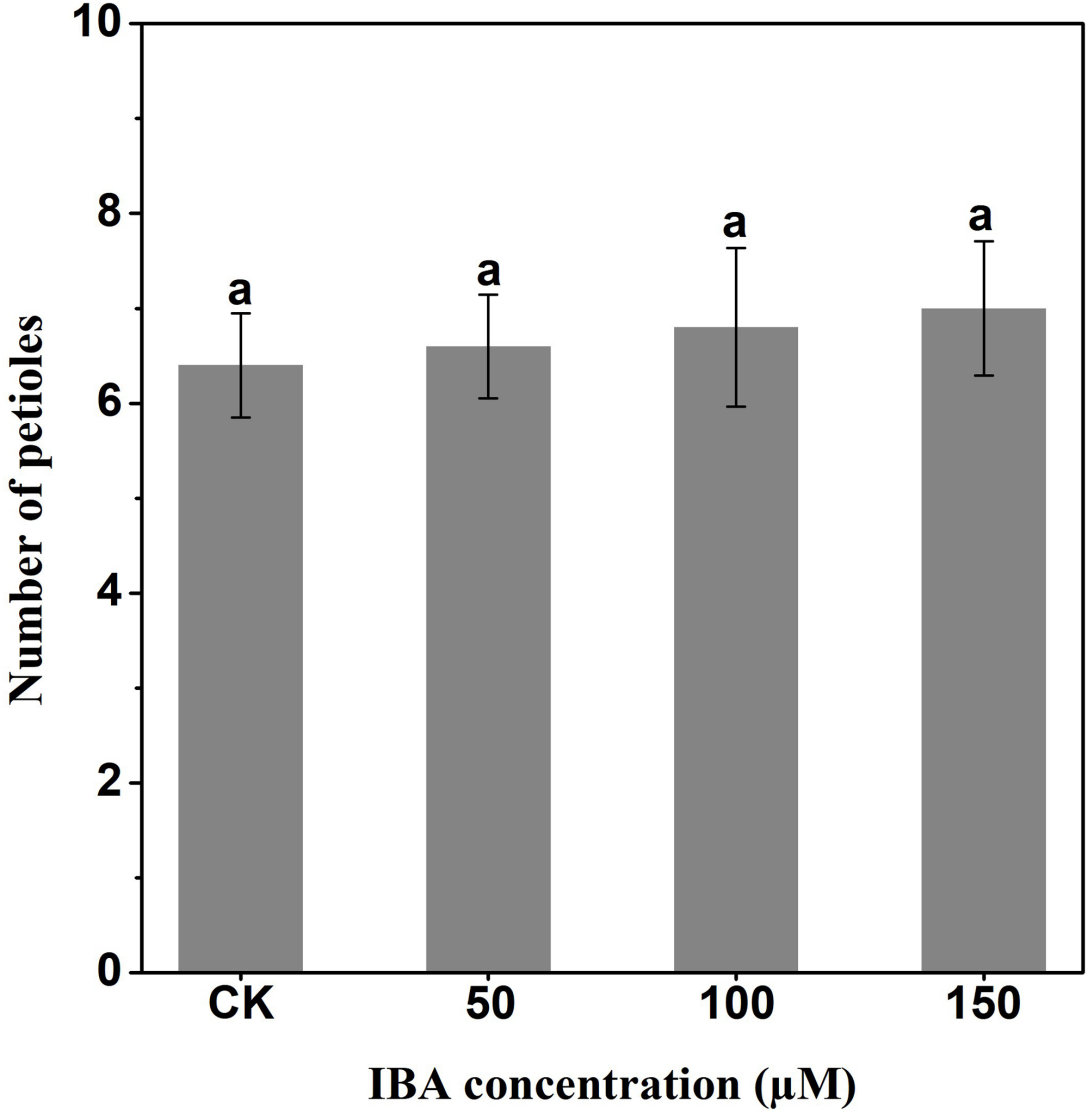
Effect of IBA on the number of petioles of carrot plants. Error bars represent standard deviation (SD). Lowercase letters represent significant differences at *P* < 0.05.

IBA significantly increased the overall plant length. The highest plant length was recorded in 150 µM IBA treatment, followed by the seedlings treated with 100 or 50 µM of IBA, respectively. Similarly, IBA treatment promoted shoot length. The highest shoot length was observed under 150 µM IBA treatment (Figs. 1 and 2). However, there was no significant difference in the number of petioles in carrot plants after IBA application (Fig. 3).

**Figure 4:**
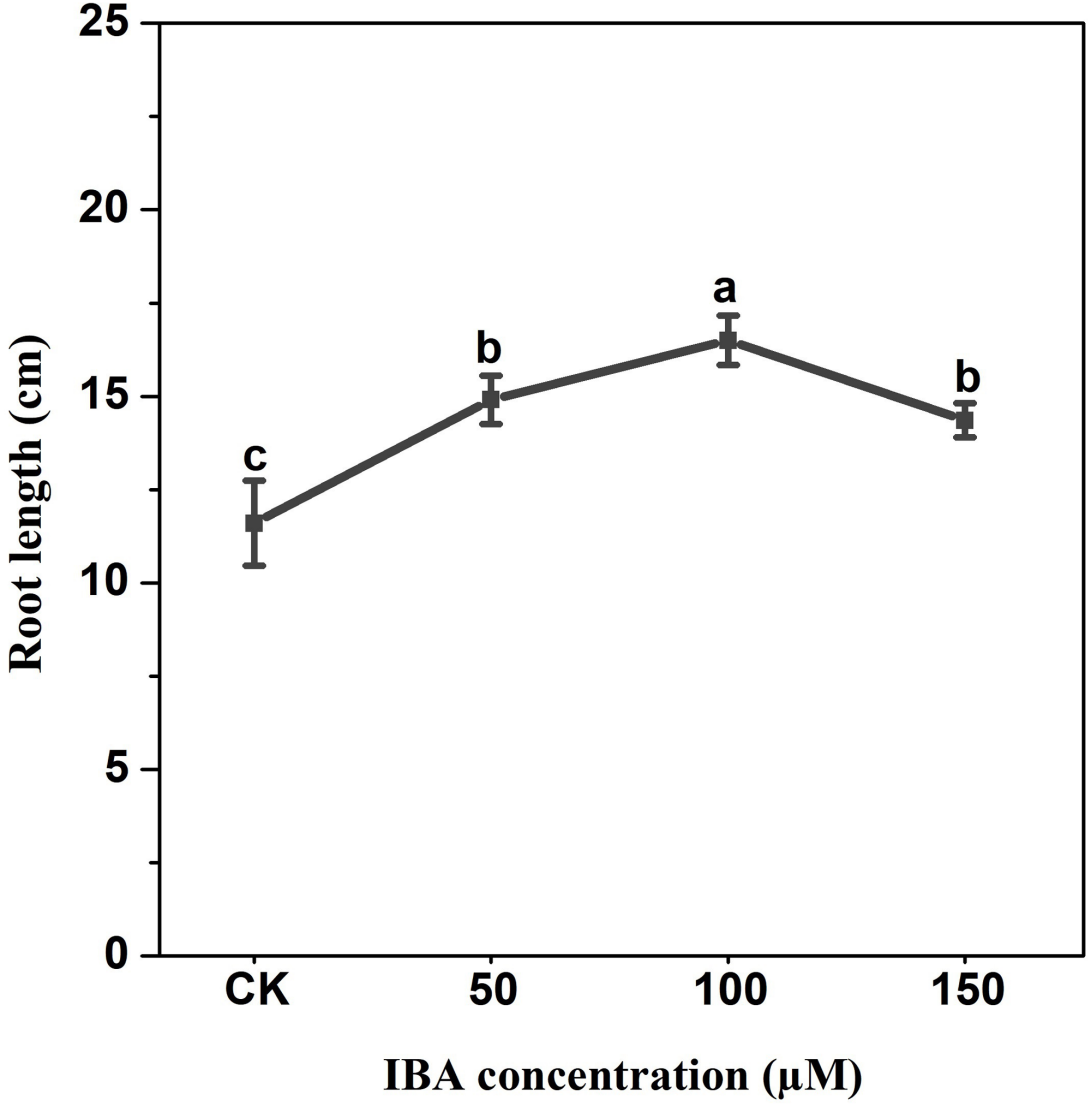
Effect of IBA treatment on carrot root length. Error bars represent standard deviation (SD). Lowercase letters represent significant differences at *P* < 0.05.

**Figure 5:**
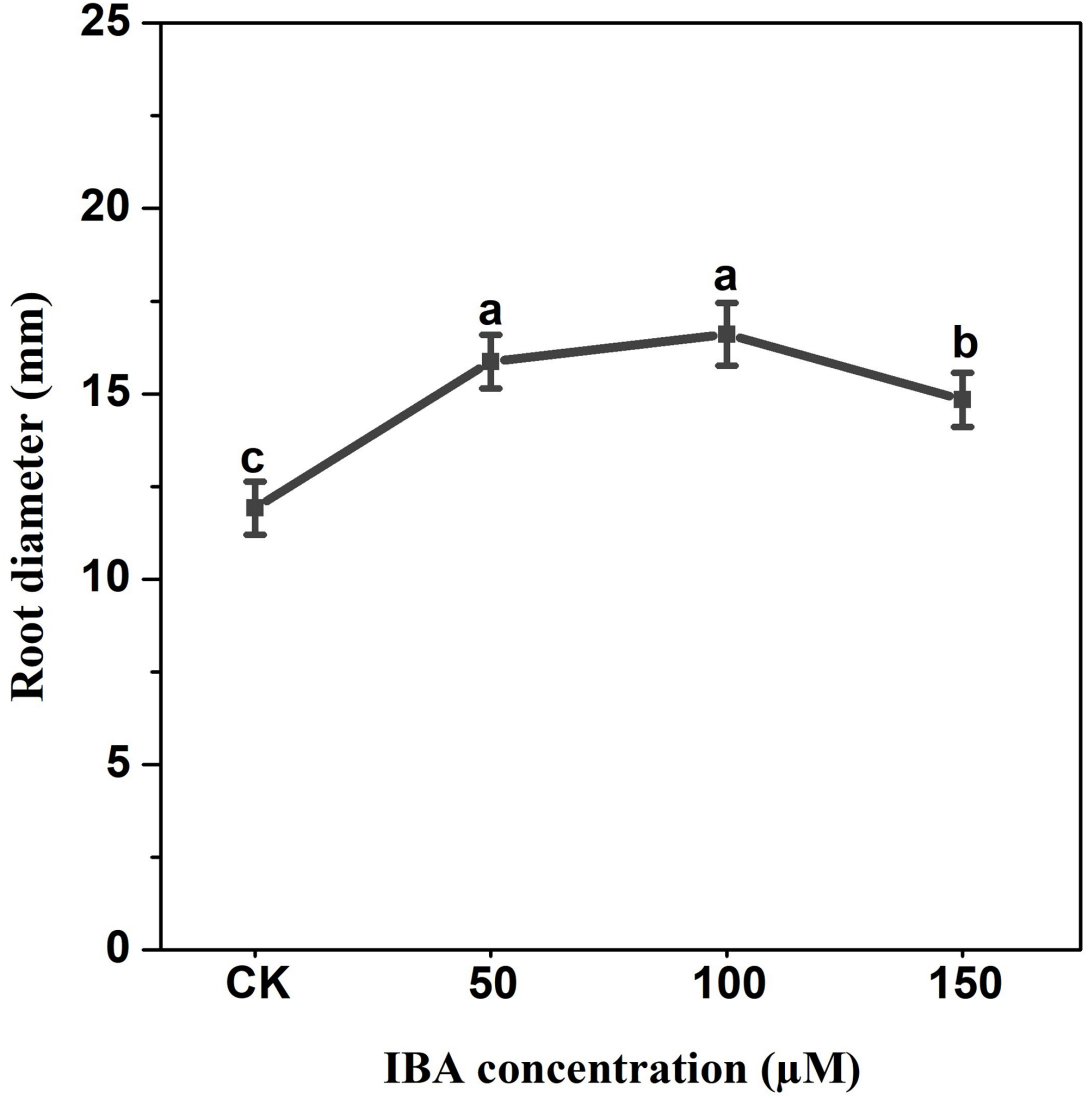
Effect of IBA treatment on carrot root diameter. Error bars represent standard deviation (SD). Lowercase letters represent significant differences at *P* < 0.05.

**Figure 6:**
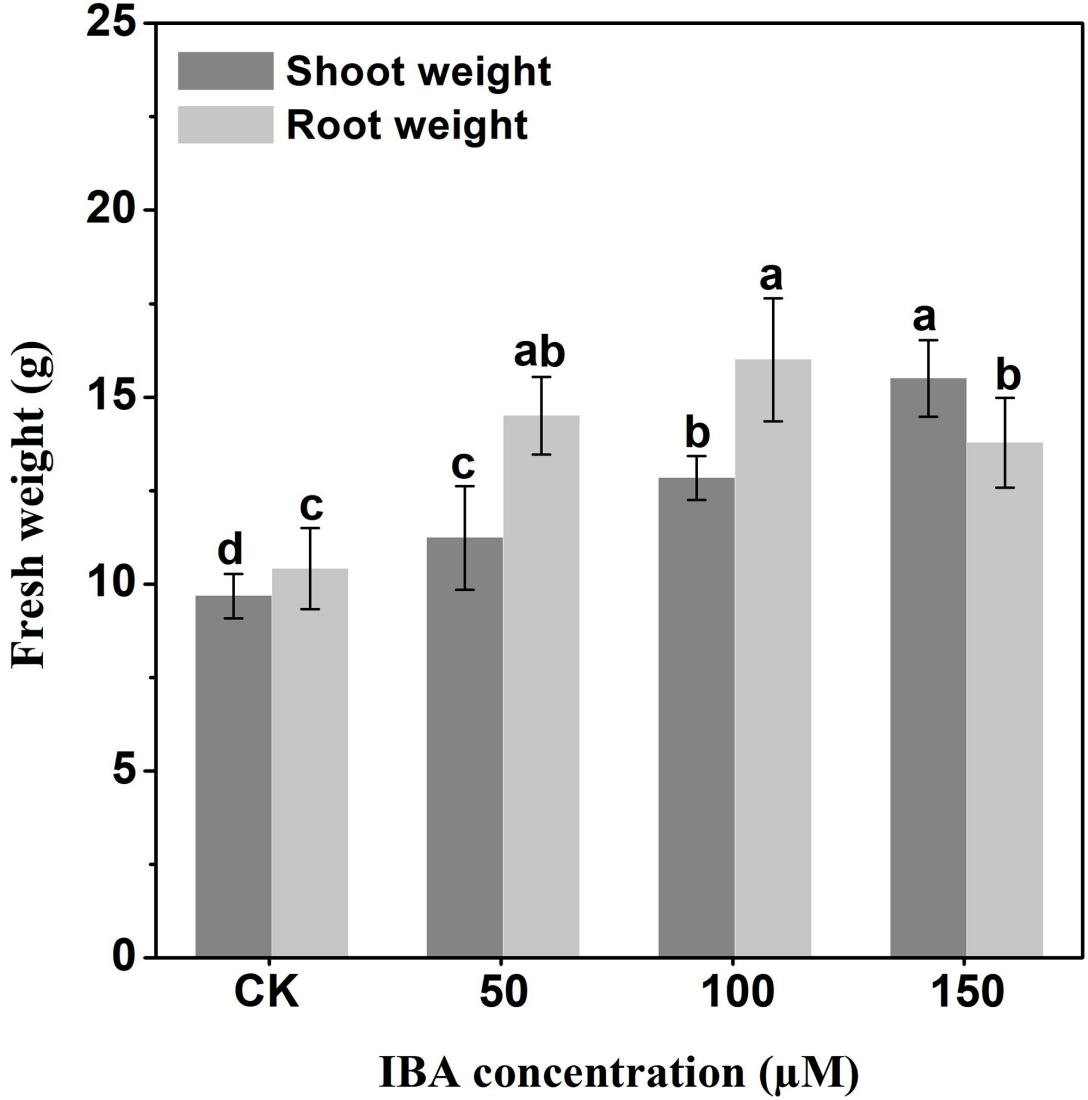
Effect of IBA on shoot and root fresh weight in carrot. Error bars represent standard deviation (SD). Lowercase letters represent significant differences at *P* < 0.05.

IBA application enhanced the taproot length of carrot. The highest taproot length was recorded under 100 µM IBA (Fig. 4). IBA concentrations of 50, 100, or 150 µM significantly increased the diameter of carrot taproot as compared with control group (Fig. 5). Further, IBA significantly increased the fresh weight of both shoot and taproot as compared with control group (Fig. 6). 150 µM IBA treatment induced the highest increase in the shoot fresh weight. The taproot fresh weight was increased by 53%, 39%, and 32%, respectively, after 100, 50, and 150 µM IBA treatment.

### Effect of IBA on the anatomical structure of carrot taproots

**Figure 7:**
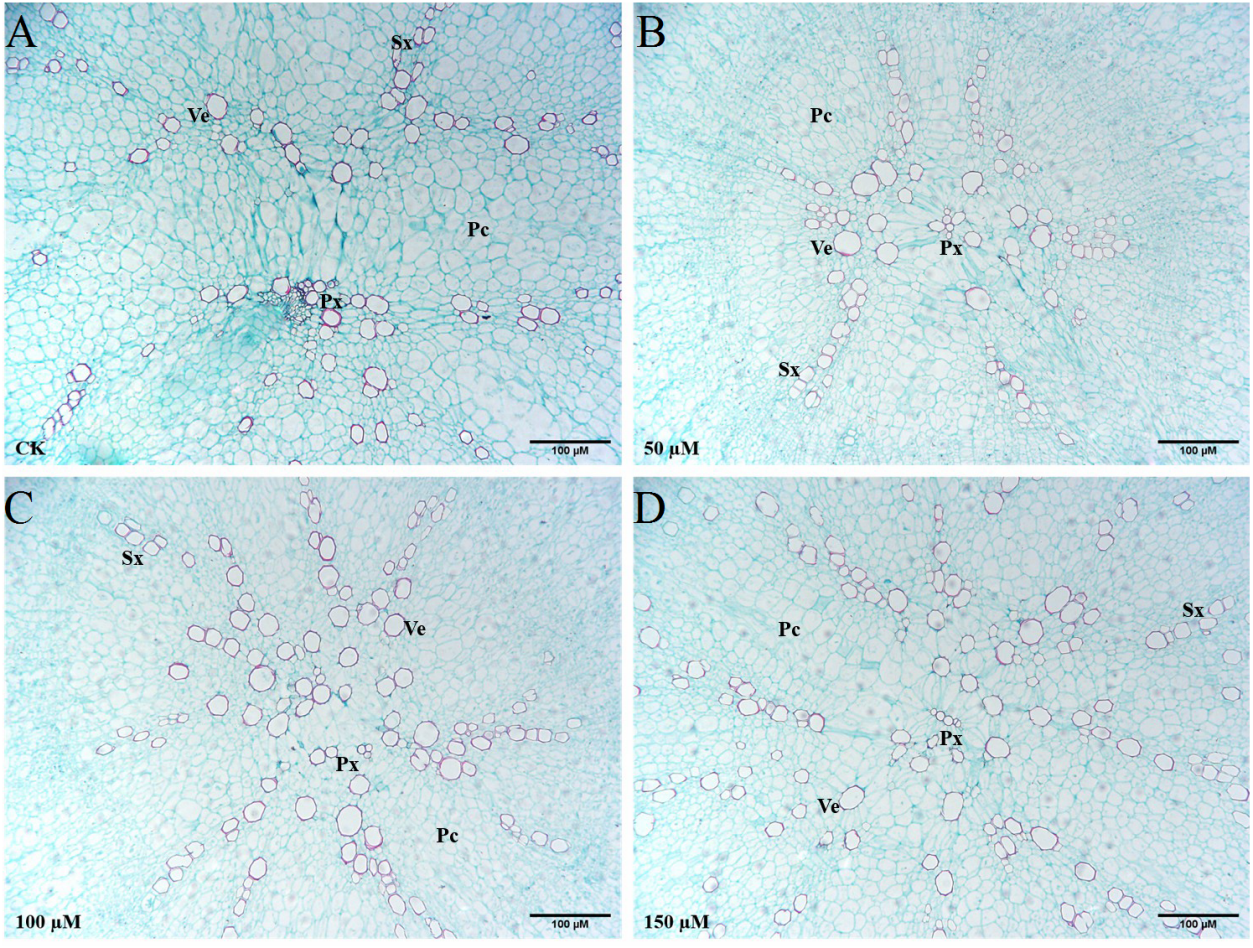
Influence of IBA on the anatomical structure of carrot roots. The central part of the root cross-sections treated with 0 (A), 50 (B), 100 (C), and 150 (D) µM IBA were shown. PC, parenchymal cell; Px, protoxylem; SX, secondary xylem; Ve, vessel. Scale bars in the lower right corner represent 100µm in length.

The transverse sections of taproots treated with different concentrations of IBA were taken and stained with safranin *O* and fast green to highlight the basic anatomical structure (Fig. 7). Exogenous application of 100 or 150 µM IBA increased the xylem area. There was no significant difference in the xylem area between the 50 µM treatment and control group. Compared with the control group, the number of xylem vessels was the highest in response to 150 µM IBA application. In addition, it was noticed that the doses of 100 and 150 µM enhanced the diameter of xylem vessels in comparison with the control group.

### Effect of IBA application on lignin accumulation in carrot taproots

**Figure 8:**
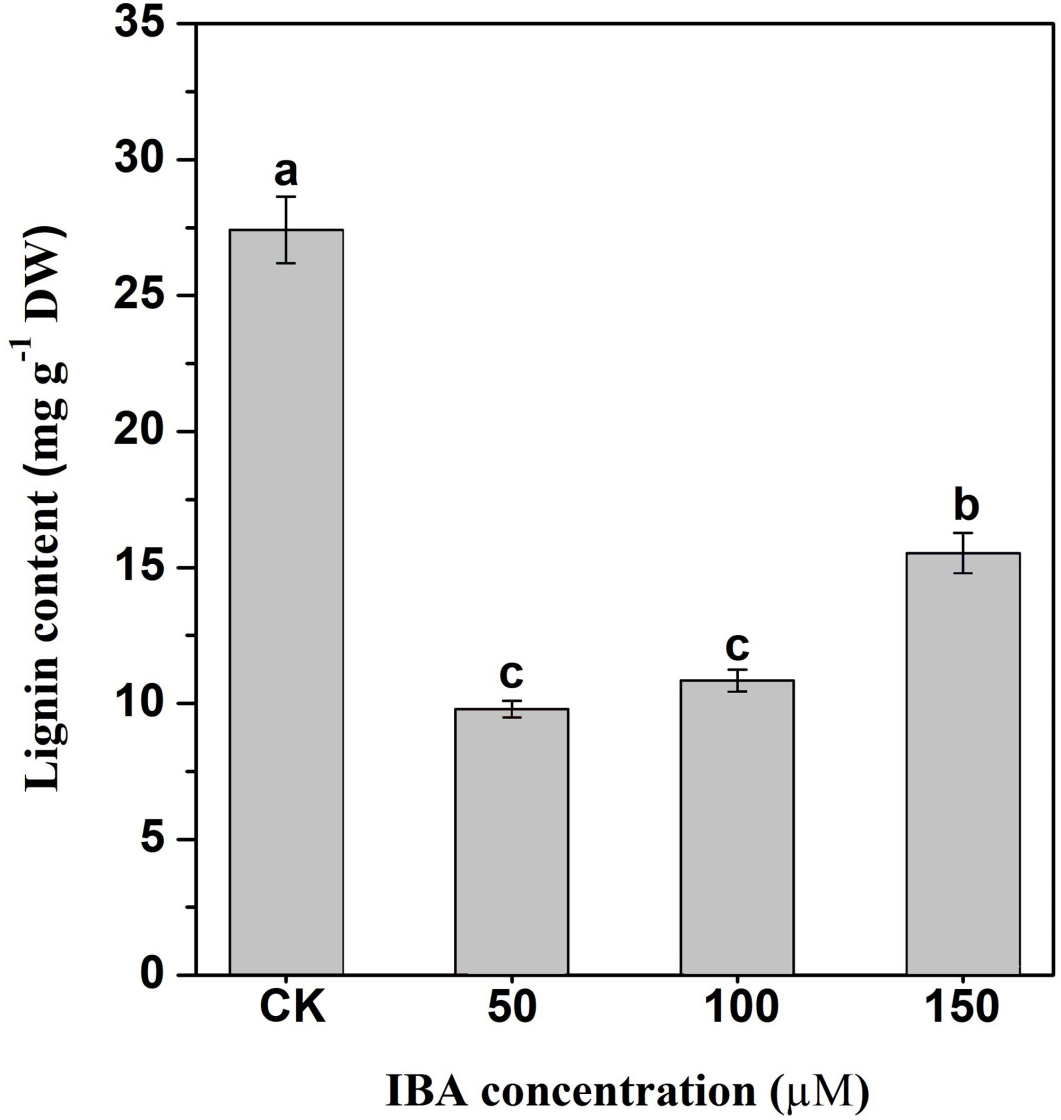
Influence of IBA on lignin accumulation in carrot roots. Error bars represent standard deviation (SD). Lowercase letters represent significant differences at *P* < 0.05.

Lignin accumulation in carrot taproots under different treatments was determined. The application of IBA significantly decreased lignin content in carrot taproots (Fig. 8). The lignin content in the control group was about 27.4 mg g^−1^. The lignin levels were about 9.8, 10.9, and 15.5 mg g^−1^ in taproots treated with 50, 100, and 150 µM IBA, respectively.

### Influence of IBA on expression profiles of key genes involved in lignin biosynthesis and polymerization

**Figure 9:**
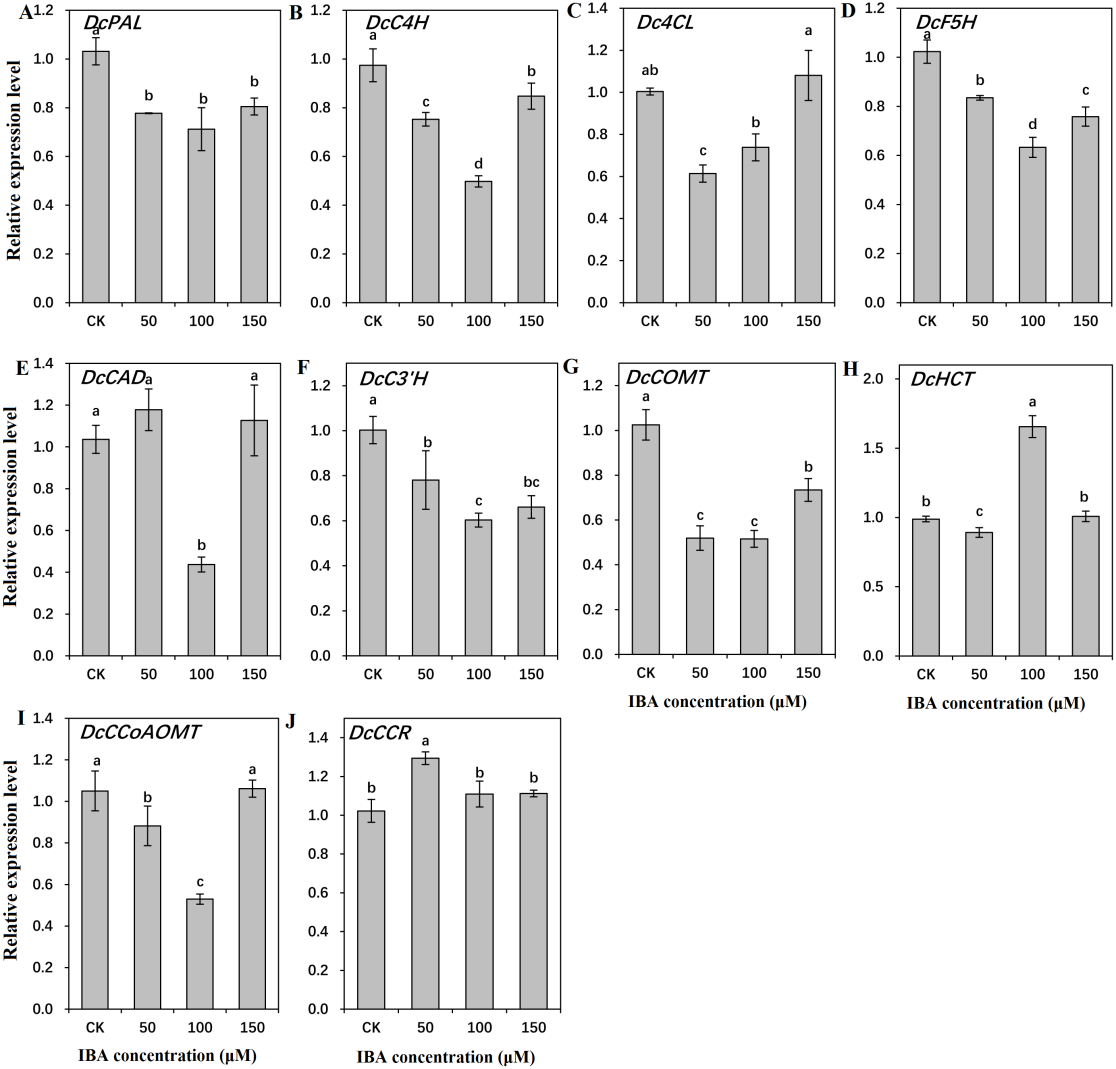
Effect of IBA on the expression profiles of genes involved in lignin biosynthesis in carrot roots. The genes encoding ten key enzymes in lignin biosynthesis are *DcPAL* (A), *DcC4H* (B), *Dc4CL* (C), *DcF5H* (D), *DcCAD* (E), *DcC3’H* (F), *DcHCT* (G), *DcCOMT* (H), *DcCCoAOMT* (I), and *DcCCR* (J). Error bars represent the standard deviation (SD) of three replicates. Data are the mean ±  SD of three independent replicates. Lowercase letters represent significant differences at *P* < 0.05.

**Figure 10:**
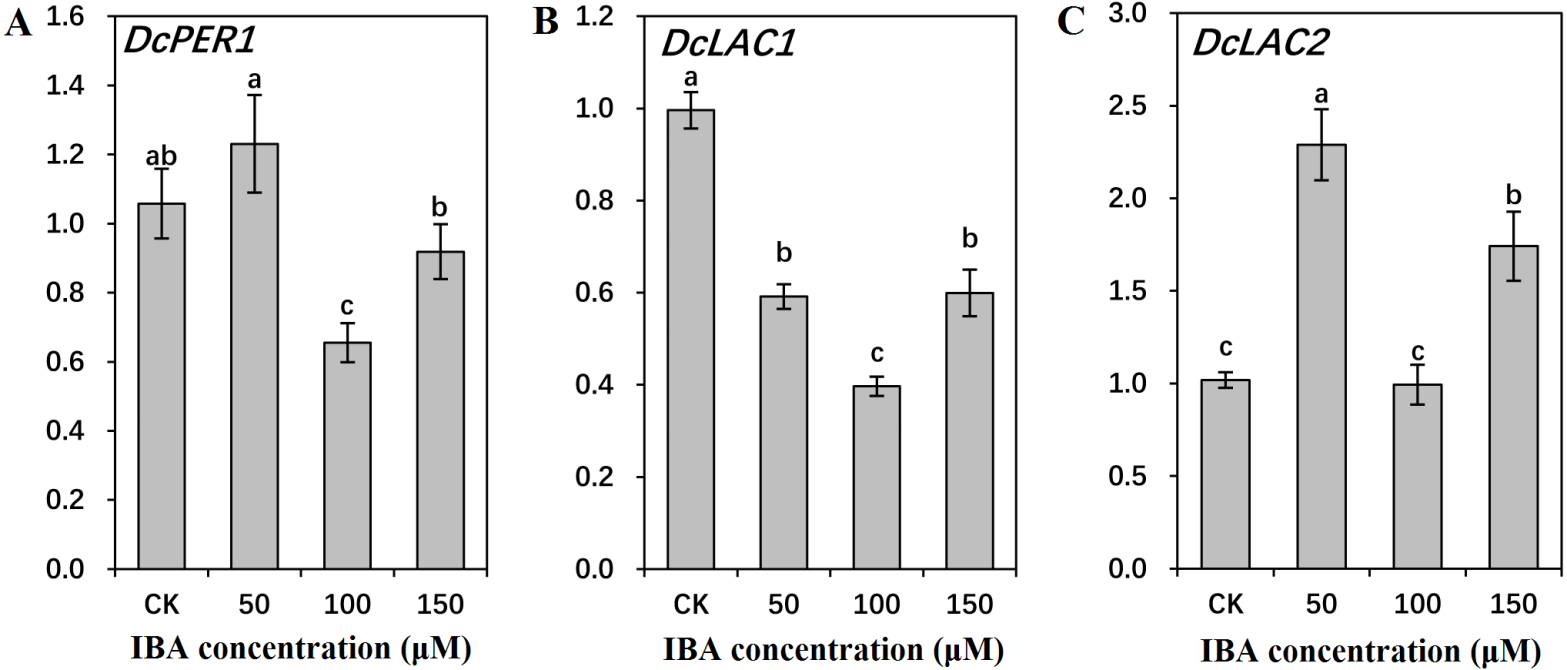
Effect of IBA on the expression profiles of genes involved in lignin polymerization in carrot roots. The genes involved in lignin polymerization are *DcPER1* (A), *DcLAC1* (B), and *DcLAC2* (C). Error bars represent the standard deviation (SD) of three replicates. Data are the mean ±  SD of three independent replicates. Lowercase letters represent significant differences at *P* < 0.05.

To find out the molecular mechanisms underlying the role of IBA in lignin accumulation in carrot taproots, transcription of key genes involved in lignin biosynthesis and polymerization were detected. As shown in Figs. 9 and 10, expression patterns of most lignin-related genes including *DcPAL*, *DcC4H*, *Dc4CL*, *DcF5H*, *DcC3′H*, *DcCOMT*, *DcCCoAOMT*, and *DcLAC1* were significantly decreased under IBA treatment.

Transcription of *DcPAL* underwent an evident decline when exposed to IBA treatment. *DcC4H* presented a tendency of decrease in mRNA abundance after 50 and 100 µM IBA treatment, and was highly expressed after 150 µM treatment. *Dc4CL* and *DcCCoAOMT* expression underwent a sharp decline after 50 and 100 µM IBA treatment, whereas no obvious difference was detected after 150 µM treatment. *DcHCT* was lowly expressed after 50 µM IBA treatment, and showed the highest expression in plants treated with 100 µM IBA treatment. Transcript levels of *DcF5H*, *DcCAD*, *DcC3′H*, and *DcCOMT* were the lowest in 100 µM IBA treatment. Expression of *DcCCR* displayed the highest expression after 50 µM IBA treatment, and reduced when a higher concentration of IBA was applied (Fig. 9).

Both 50 and 150 µM IBA treatment had no obvious effect on *DcPER1* expression, whereas 100 µM IBA induced a sharp declined in *DcPER1* mRNA abundance. Transcription of *DcLAC1* underwent a constant decline after 50 and 100 µM IBA treatment, and increased after 150 µM treatment. By contrast, 50 and 150 µM IBA treatment increased *DcLAC2* expression as compared with control group (Fig. 10).

## Discussion

Auxins are organic substances, which promote the growth and development of plants at low concentrations. Auxins regulate numerous developmental processes in plants including cell expansion, root initiation, vascular tissue differentiation, bud and flower growth (Davies, 2013). Auxin and cytokinin are the main phytohormones that control root growth, root gravitropism, and vascular differentiation (Aloni et al., 2006).

Carrot has high nutritional values, and is one of the most important vegetable crops grown throughout the world. The fast growth of the food industry has led to increased carrot consumption (Wang et al., 2018b; Que et al., 2019). In the current study, carrot taproots were treated with different concentrations of IBA to study the effect of IBA on carrot taproot growth and development. The roles of IBA in regulating expression of lignin-related genes and lignin accumulation in carrot taproot are still unknown. The present study results could enhance the understanding of the roles of IBA in regulating growth and taproot lignification in carrot, particularly at the molecular level.

Our results showed that IBA treatment increased carrot plant length, shoot length, and shoot weight. In contrast, there was no significant effect on the number of petioles after IBA treatment as compared with control group. This increase may be attributed to the role of auxin in stimulating cell division and elongation (Davies, 2013). IBA application was able to induce the elongation of the internodes, leading to the promotion of stem growth of pea plants (Yang & Davies, 1999). Also, IBA application increased the leaf area and dry weight of leaves in chickpea (Amin et al., 2013). Thus, the increase in shoot fresh weight after IBA treatment may result from the increase in shoot length, leaf area, and leaf fresh weight. These results are consistent with previous studies which observed that the application of IBA promoted plant and shoot length, plant height, and dry weight of both branches and leaves (Amin et al., 2013; Hayat et al., 2009). IBA significantly enhanced taproot length, diameter, and fresh weight, and the highest increase was observed in the 100 µM IBA treatment. Similar results were obtained in maize; low concentrations of IBA (10^−9^ M∼10^−12^ M) significantly increased the length, root diameter and cortex thickness of the primary root (Šípošová et al., 2019). They also demonstrated that high concentration of IBA (10^−7^ M) decreased the primary root length. High doses of auxin restrict root growth *via* proton pump inhibition and lead to alkalinization of the cell wall, whereas low doses ensure the acidification of apoplast and activate the vital wall-loosening proteins (Evans, Mulkey & Vesper, 1980).

Auxin is a kind of plant hormones that regulate the development of the root. It can stimulate xylem tissue differentiation (Wareing, 1958; Aloni et al., 2006). Exogenous IAA application induced the cambial division, and thus promoted xylem tissue differentiation (Wareing, 1958; Digby & Wareing, 1966). Auxin stimulated the elongation of vessels in *Populus robusta*, and improved the radial diameter of xylem elements (Digby & Wareing, 1966). In *Capsicum* and *Solanum*, auxin boosted the growth of secondary xylem, whereas auxin inhibitors fully or partially displayed the contrary traits (Samantarai & Nanda, 1979). Here, we detected an increase in number, diameter and area of xylem vessels in response to IBA (100 and 150 µM) treatment.

According to the previous studies, lignin deposition mainly exists in the walls of thickened cells (Rogers & Campbell, 2004; Zhao & Dixon, 2011). Lignin is an essential component of the plant cell wall. The primary function of lignin is to provide mechanical support and water transport (Somerville et al., 2004). However, high deposition hampers the taste, texture and quality of carrot taproot, whereas the decline in lignin content may affect plant growth and development (Bonawitz & Chapple, 2010). IBA treatment decreased lignin content of carrot in a dose-dependent manner. This finding is in agreement with the results of the previous study, which demonstrated IBA inhibited hypocotyl lignification in soybean (Chao et al., 2001). Similar results were observed when soybean hypocotyls were treated with NAA (Chen et al., 2002). Also, it was observed that IBA-treated *Cinnamomum kanehirae* cuttings exhibited a decrease in lignin content (Cho et al., 2011).

Previous studies have suggested that changes in lignin levels were attributed to altered expression of the genes in the lignin biosynthesis pathway (Ali & McNear, 2014; Wang et al., 2017; Duan et al., 2019). In the current study, most of the studied genes (*DcPAL*, *DcC4H*, *Dc4CL*, *DcF5H*, *DcC3′H*, *DcCOMT*, *DcCCoAOMT*, and *DcLAC1*) in the lignin synthesis pathway exhibited decreased expression in response to IBA treatment. Our earlier reports found that the decreased lignin accumulation was related to the lower expression level of key genes in the lignin biosynthesis pathway. The declined expression levels of *PAL*, *HCT*, *CCR*, *CAD* and *C3′H* genes exhibited an impact on lignin accumulation (Baucher et al., 2003; Chen et al., 2006; Ralph et al., 2006; Wagner et al., 2007; Derikvand et al., 2008; Wadenbäck et al., 2008). In alfalfa and tobacco, repression of genes in the pathway, such as *PAL*, *C4H*, *HCT* and *C3′H,* caused a decrease in total lignin levels (Li, Weng & Chapple, 2008). Moreover, the decline in *HCT* gene expression resulted in a significant reduction in lignin content and changed lignin composition (Li, Weng & Chapple, 2008). Likewise, the mutants *4cl1*, *c4h* and *ccr1* showed lower lignin content than that of the wild type (Vanholme et al., 2012). In *Populus tremuloides*, inhibition of *4CL1* resulted in a 45% reduction in lignin content (Hu et al., 1999). However, inhibition of *F5H* that is involved in S lignin synthesis, leads to the change of S/G ratio with only minor influence on content of total lignin (Li, Weng & Chapple, 2008). In *Arabidopsis*, the mutant *f5h1* showed an obvious decrease in the S unit, whereas no change was determined in the overall lignin levels (Vanholme et al., 2012). Auxin inhibited peroxidase gene expression *via* regulating various auxin responsive elements in the promoter of the peroxidase genes (Klotz & Lagrimini, 1996; Chou, Huang & Liu, 2010). Exogenous application of IBA decreased lignin content, which was accompanied by repressed peroxidase gene expression and peroxidase activity in soybean hypocotyl and *Cinnamomum kanehirae* cuttings (Chao et al., 2001; Cho et al., 2011).

## Conclusions

IBA application significantly improved the growth parameters of carrot. 100 or 150 µM IBA treatment increased the number and area of xylem vessels, whereas declined lignin levels were detected in IBA-treated carrot plants. Furthermore, the expression levels of lignin-related genes were significantly decreased in response to IBA treatment. Our results could improve understanding of the roles of IBA in carrot taproot growth and lignin accumulation, especially at the molecular level.

##  Supplemental Information

10.7717/peerj.10492/supp-1 Supplemental Information 1 Raw data for growth characteristics, lignin accumulation, and gene expression

## Abbreviations

4CL: 4-coumarate-CoA ligase
C3’H: p-coumaroyl shikimate/quinate 3-hydroxylase;
C4H: Cinnamate 4-hydroxylase;
CAD: Cinnamyl alcohol dehydrogenase
CCoAOMT: Caffeoyl-CoA O-methyltransferase
CCR: Cinnamoyl-CoA reductase
COMT: Caffeic acid O-methyltransferase
DW: Dry weight
F5H: Ferulate 5-hydroxylase
HCT: Hydroxycinnamoyl-CoA shikimate/quinate
IAA: Indole-3-acetic acid
IBA: Indole-3-butyric acid
LAC: Laccase
NAA: Naphthalene acetic acid
PAL: Phenylalanine ammonia lyase
PER: Peroxidase
RT-qPCR: Real-time quantitative PCR

## Additional Information and Declarations

### Data Availability

The following information was supplied regarding data availability: The raw data for plant morphological characteristics, absorbance values for lignin determination, and the RT-qPCR experiment are available in a Supplemental File.
